# Editorial: Maternal psychopathology in pregnancy and the postpartum period and its impact on infant and child development

**DOI:** 10.3389/fpsyg.2025.1672807

**Published:** 2025-10-03

**Authors:** Ljiljana Jeličić, Sarah Nazzari, Mile Vuković

**Affiliations:** ^1^Cognitive Neuroscience Department, Research and Development Institute “Life Activities Advancement Institute”, Belgrade, Serbia; ^2^Department of Brain and Behavioral Sciences, University of Pavia, Pavia, Italy; ^3^Faculty of Special Education and Rehabilitation, University of Belgrade, Belgrade, Serbia

**Keywords:** maternal mental health, perinatal depression, intergenerational risk, infant development, family systems, postpartum period

Maternal mental health is increasingly recognized as a significant public health concern. A substantial proportion of pregnant women experience psychological difficulties—most commonly stress, anxiety, and depression. However, these conditions often remain undetected and unaddressed in clinical settings, despite their substantial health and social impact and their association with adverse child outcomes (Jimènez-Barragan et al., 2024). There is growing evidence that maternal psychological states during pregnancy not only impact maternal wellbeing but are also closely associated with infant behavioral outcomes and may represent key determinants of early neurodevelopment (Wu et al., 2024; Jagtap et al., 2023; Severo et al., 2023). Theoretical frameworks, including the fetal programming hypothesis (Barker and Osmond, 1986), suggest that maternal psychological states during pregnancy may influence fetal development through subtle alterations in the intrauterine environment, potentially contributing to later physical and mental health outcomes (Van den Bergh et al., 2018; Phua et al., 2023; Jagtap et al., 2023).

Given the multifactorial nature of maternal and neonatal health—including biological, psychological, and environmental factors across the perinatal period—ongoing scientific efforts are essential to deepen our understanding of this complex yet critical domain of developmental and preventive health. The articles included in this collection contribute to this goal by examining key aspects of maternal mental health and its impact on both maternal wellbeing and offspring development, using diverse methodologies and interdisciplinary perspectives.

This Research Topic, “*Maternal Psychopathology in Pregnancy and the Postpartum Period and Its Impact on Infant and Child Development,”* features eight papers—six original quantitative studies and two reviews—that explore associations between parental mental health, parenting behaviors, and developmental outcomes in infants and children. Among the original studies, one employed an experimental design, four used longitudinal designs, one used a cross-sectional approach, and one was a single-arm feasibility study. All studies predominantly involved maternal participants, reflecting the central role of mothers in perinatal and early developmental research. Several investigations included mother-infant dyads, enabling direct assessments of early relational dynamics. Additionally, some studies incorporated grandparents, acknowledging the broader familial context that shapes maternal mental health and child development. One study further included fathers and adolescent offspring, emphasizing the intergenerational transmission of mental health trajectories.

This diverse range of participant groups underscores the complexity of family systems and the importance of adopting comprehensive, multi-informant approaches to understanding parental influences across the lifespan. Most studies were conducted in Italy, one in China, and the two review articles covered international literature. Together, the findings offer valuable insights across varied cultural contexts. Key themes from the collection are summarized below.

## Maternal mental health and early parent-infant relationships

Several studies emphasize the pivotal role of maternal mental health during pregnancy and the postpartum period in shaping early attachment and caregiving quality. For example, Carone et al. show that higher prenatal attachment coherence and adaptive defensive functioning predict stronger mother-infant relationships at 6 months postpartum. Complementing this, work by Arioli et al. suggest that interventions aimed at reducing prenatal anxiety and enhancing maternal-fetal attachment—whether through passive relaxation or active engagement protocols—demonstrate promise in improving maternal wellbeing and perinatal outcomes. Collectively, these findings highlight the importance of supporting maternal emotional regulation and attachment capacities to foster healthy early relational dynamics.

## Intergenerational transmission of mental health risks

Longitudinal data highlight the intergenerational effects of parental mental health. Notably, in Zhai and Yang's work, both maternal and paternal depression trajectories significantly predict depressive symptoms in adolescence, highlighting the multifactorial influences within family systems, including both genetic and environmental contribution. Furthermore, Sperati et al. show that maternal perinatal depression can influence infant temperament, increasing negative affect and fear responses in early infancy. These findings suggest that addressing parental mental health during the perinatal period has implications that extend well beyond infancy, influencing long-term developmental trajectories.

## Social support and family context

Social support emerges as a crucial protective factor for maternal mental health. Work by Sperati et al. show that partner support during pregnancy is associated with lower maternal depressive symptoms, while work by Riem and van der Straten reviewed emerging evidence that highlights grandparental support as a potentially valuable yet underexplored contributor to maternal and child wellbeing. These findings underscore the need to consider broader familial networks in the design of perinatal mental health interventions.

## Measurement tools and healthcare quality

The validation of novel tools, such as the Disrespect and Mistreatment during Childbirth Questionnaire (DMCQ), as provided by work by Suttora et al., offers insight into the psychosocial dimensions of childbirth and their impact on parenting stress. This instrument captures key aspects of obstetric care quality, linking negative healthcare experiences to heightened postpartum distress. In addition, Lega et al. show in an implementation study of arts-based interventions, such as group singing, the feasibility and acceptability of innovative, non-medicalized approaches to supporting mothers with postpartum depression. Lastly, Yang et al. provide a timely review of emerging treatments for perinatal depression, including pharmacological options and natural supplements. While interventions like saffron and vitamin D show promise, the authors stress the need for rigorous trials to validate their safety and efficacy, particularly for breastfeeding mothers.

## Gaps in knowledge and future directions

Despite significant advances, critical gaps persist in the field of maternal psychopathology. While some studies demonstrate methodological strengths, including longitudinal designs and the inclusion of fathers and grandparents, important limitations remain, particularly the reliance on maternal self-report and cultural homogeneity in predominantly Western samples. Additional gaps include limited attention to broader family dynamics, unclear mechanisms underlying intergenerational transmission, and insufficient research on long-term outcomes and complementary interventions. Addressing these gaps requires multidisciplinary, culturally sensitive, and longitudinal approaches, with a focus on early identification and tailored support targeting maternal attachment, emotional regulation, and social support. Expanding research to include diverse family members and extending follow-up into adolescence will deepen understanding of developmental trajectories and guide comprehensive prevention and intervention strategies. Importantly, this evidence may also have significant implications for clinical practice and service delivery. Awareness of maternal psychopathology and its impact on child development can guide early screening and timely identification of at-risk mothers. Engaging fathers and other family members in preventive and therapeutic strategies may further enhance outcomes. Together, these insights can promote multidisciplinary, culturally sensitive approaches that improve accessibility and effectiveness of perinatal mental health care.
